# Volatile Characterization of Recovery Minority Grape Varieties from Castilla-La Mancha Region (Spain)

**DOI:** 10.3390/plants13111507

**Published:** 2024-05-30

**Authors:** Cristina Cebrián-Tarancón, Argimiro Sergio Serrano, Juan L. Chacón-Vozmediano, Jesús Martínez-Gascueña, Gonzalo Alonso

**Affiliations:** 1 Department of Agricultural Chemistry, School of Agricultural and Forestry Engineering and Biotechnology, University of Castilla-La Mancha, Avda. de España s/n, 02071 Albacete, Spain; cristina.ctarancon@uclm.es (C.C.-T.); sergio.serrano@uclm.es (A.S.S.); 2 Regional Institute of Agri-Food and Forestry Research and Development of Castilla-La Mancha (IRIAF), Ctra. Toledo-Albacete s/n, 13700 Tomelloso, Spain; jlchacon@jccm.es (J.L.C.-V.); jmartinezg@jccm.es (J.M.-G.)

**Keywords:** recovered grapevine cultivars, aroma compounds, aroma potential, water stress

## Abstract

Nowadays, the identification and characterization of grapevine cultivars resilient to climate and water stress while preserving quality traits is crucial for the wine industry. Therefore, the objective of this work was to characterize according to their aromatic potential nine white and six red minority cultivars recently recovered from Castilla-La Mancha region (Spain), subjected to two different water-deficit regimes: rainfed, with subsistence irrigation, and irrigated. For this, the varietal aromatic potential index (IPAv) and the detailed aromatic composition were analyzed via HS-SBSE-GC/MS in extracts of two different pHs. For IPAv values, red varieties did not show a clear trend with respect to irrigation. However, in white minority varieties, higher values were obtained under irrigation conditions. Thus, a clear differentiation of the minority varieties in comparison to the references was observed, primarily attributed to the content of esters and acids, in both white and red varieties. A notable contrast was observed at different pHs, indicating a greater extractability of certain compounds like linalool, under more acidic conditions. This suggests that some recovered minority cultivars could be promising for cultivation in semi-arid regions with limited water, contributing to the sustainability of the wine sector in the future.

## 1. Introduction

In recent years, viticulture has suffered significant challenges due to climate change, which has significantly transformed the climatic conditions traditionally associated with grape production. Rising temperatures, extreme weather events, and changes in rainfall patterns are altering the viticultural landscape, affecting both the quality and quantity of harvests. Historic wine-growing regions are compelled to reconsider their agricultural practices and grape varieties, aiming to adapt to an ever-evolving environment. Thus, the need for sustainable management, innovation in cultivation techniques, and the identification of grape varieties better adapted to heat and drought are becoming crucial for the preservation and ongoing success of the wine industry in this new climatic scenario. 

In this context, the recovery of minority varieties is a topic of great relevance for the sector. As industrialization and the standardization of crops progress, many traditional plant varieties have been marginalized and some of them are even on the verge of extinction. However, these varieties often possess unique characteristics and specific adaptations to their development environment, making them valuable from an agronomic perspective in ensuring resilience against climate change. In this line, the application of advanced genomic sequencing techniques has enabled researchers to identify and analyze specific genetic markers that have allowed highlighting the rich genetic diversity of recovered varieties, which could make them able to adapt to different weather conditions, resist diseases, and improve the quality of wines [1,2,3,4,5]. Likewise, in recent years, numerous studies have been conducted to explore the agronomic adaptation of these grape varieties under different water regimes. These investigations have studied the relationship between plants and their environment by assessing stable isotope ratios, primarily focusing on carbon (δ13C) and water potentials, among other factors. The findings from these studies highlight the capability of certain cultivars to effectively confront and adapt to diverse climatic conditions while maintaining yields and general enological parameters [6,7,8,9].

Regarding the chemical composition of grapes, understanding the impact of water stress on the phenolic and aromatic composition, both free and bound, of these recovered minority grapes is of great importance, as these compounds play a crucial role in the quality and organoleptic characteristics of the wine. Moreover, it is known that the phenolic composition in grapes is significantly affected by water availability. Under drought conditions, grapevine plants tend to increase polyphenol synthesis as a defense mechanism. Water scarcity induces water stress that activates specific metabolic pathways, increasing the concentration of these compounds to protect against oxidative damage. So, the impact of irrigation on the phenolic composition of berries in traditional varieties has been thoroughly studied [9,10,11,12]. Thus, this effect on minority varieties has recently been considered [13]; the results revealed that some of the traditional varieties, cultivated under moderate and severe water stress conditions, are capable of maintaining productions with an adequate phenolic composition, similar to or even more favorable than other varieties more widespread. In the same line, various authors have observed a strong influence on the accumulation of volatile compounds in different widely cultivated varieties due to environmental factors, such as water availability, being positively or negatively affected by water stress depending on the family of compounds under study [14,15,16,17]. Nevertheless, the response of volatile composition to water deficit in a great number of recently recovered minority varieties remains unknown. However, this is a critical aspect, considering the potential value of these varieties in adapting to current and future climatic conditions. They can function as valuable resources, contributing to the diversity and sensory complexity of the wines produced.

Castilla-La Mancha, a region located in the heart of Spain, with a vineyard area of more than 450,000 ha, is considered one of the world’s great vineyards. The soils are predominantly limestone and vary in texture from sandy to clayey. It is particularly notable for its extreme continental climate, characterized by very hot summers and cold winters, with low annual rainfall, generally less than 400 mm, making drought a constant concern for winegrowers. For that, studying new varieties that are able to adapt to these new climate change situations is of vital importance for the survival of viticulture in this region. Therefore, considering all of the aforementioned factors, the aim of this work is to characterize the volatile composition under two different water regimes of 9 widespread and 15 recovered/minority grape varieties, both red and white. The grape varieties selected for this study were chosen for their historical and cultural significance in the region. These varieties, which have been cultivated for centuries, offer a rich genetic heritage that can provide valuable information on resistance to drought and other environmental stress factors.

## 2. Results and Discussion

### 2.1. Varietal Aroma Potential Index (IPAV)

Water availability is a crucial factor that influences the development of aromatic compounds in grapes. Therefore, understanding the adaptability of numerous varieties to water stress and its impact on the aromatic potential of grapes is essential. In this context, the IPAv parameter serves as an indicator of the global content of glycosylated aroma precursors in grapes, which includes mostly volatile aglycones such as alcohols, terpenes, phenols, and C13-norisoprenoids [18]. Thus, a higher value of this index indicates that grapes could have a greater amount of glycosylated aroma precursors, which could be liberated during the winemaking process, contributing positively to the aromatic characteristics of wines. The IPAv values of white and red grapes, in both water regimes, are shown in Table 1.

In spite of some papers having suggested that water deficit resulted in improved grape aroma [19], the IPAv values in white varieties were higher under the irrigation regime compared to rainfed for most varieties, showing a positive influence of irrigation on the development of glycosylated aroma precursors in grapes. In reference varieties, the higher increases between the two regimes were found in Airén and Macabeo, with values of 19.53 and 14.85, respectively, so irrigation seems to have a more pronounced impact on the aromatic potential of these grapes, which has already been pointed out by other authors [14]. Regarding recovered varieties, Moscatel Serrano showed the highest increase in IPAv values under irrigation regime, 20.51 versus 12.08 in rainfed conditions. This variety, together with Albillo Dorado, showed higher IPAv values than the reference ones, in both water regimes, which suggests that they could be a good growing alternative in these climate change conditions, resulting in wines with a different aromatic profile. By contrast, in Azargón, Jarrosuelto, or Mizancho, differences between the two regimes were less notable, so these cultivars have a moderate response to irrigation in terms of aromatic potential. Only the varieties Chardonnay and Maquías exhibited lower IPAv values under the irrigation regime compared to rainfed, 5.97 and 7.69, respectively.

In the case of red varieties, behavior opposite to white ones was observed, with IPAv values lower under the irrigation regime for most varieties, with Garnacha Tinta and Tempranillo being the reference varieties that showed the highest decrease, suggesting a sensibility particularly responsive to additional water supply, resulting in a decrease in the aromatic potential characteristics. Of the recovered varieties, Tinto Velasco was the minority red variety most influenced by the water regime, reducing its IPAv value from 13.24 to 8.48. On the other hand, Benedicto was the red cultivar that showed the highest increase in IPAv value under an irrigation regime, from 11.73 to 16.67, showing higher values than any of the reference varieties, which suggests an important aromatic potential of this variety if it is grown under irrigation conditions.

In summary, the data show that irrigation tended to increase the aromatic potential of white grape varieties. However, this tendency was not as clear for red varieties, as the magnitude of this effect varied depending on the particular variety. Even so, most of the minority varieties studied have shown IPAv values under rainfed conditions similar to, and in some cases even higher than, those of the reference varieties. Therefore, although it is important to consider the individual characteristics and responses of each grape variety when applying irrigation strategies to enhance their aromatic qualities, these results allow us to consider these varieties as a good alternative for cultivation in wine-growing regions most affected by these climate change conditions.

### 2.2. Volatile Composition of Grapes

**Figure 1:**
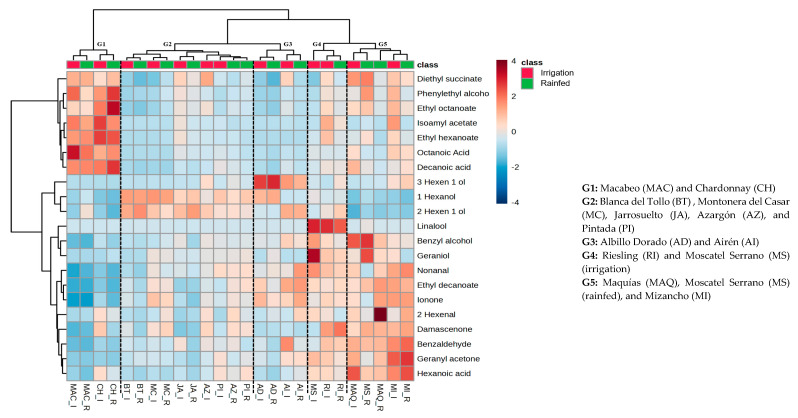
Heatmaps of white grapes’ aromatic compounds at extract pH 3.6.

**Figure 2:**
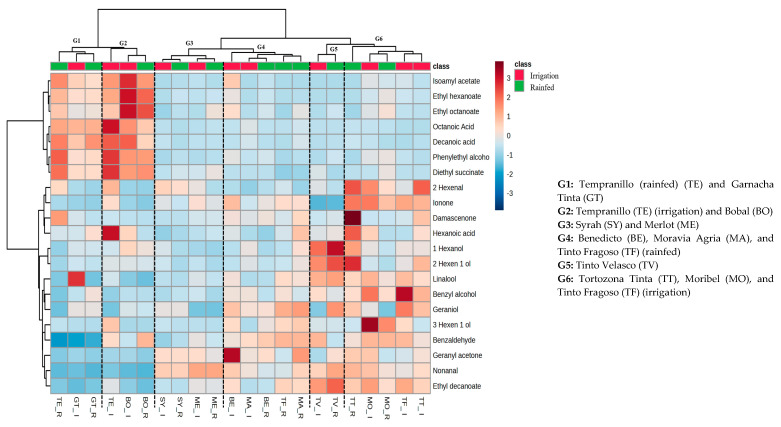
Heatmaps of red grapes’ aromatic compounds at extract pH 3.6.

In order to know the similarity and correlation between the aromatic profile of reference and minority varieties, a heatmap with cluster analysis was carried out for white and red grapes. In this way, the smaller the distance separating two clusters, the greater the similarity between the samples contained within these clusters. So, Figure 1 and Figure 2 show the results obtained for the white and red varieties, respectively. In the case of white grapes, we can distinguish two well-differentiated clusters. One of the clusters contains a single group (G1) with the reference varieties Macabeo and Chardonnay in both water regimes. The second cluster contains four groups (G2, G3, G4, and G5) with all minority varieties and the rest of the reference varieties. In the same way, in the case of red grapes another two main clusters are also differentiated. In the first, we find two groups (G1 and G2), which encompass the traditional varieties Tempranillo, Garnacha Tinta, and Bobal. In the second main cluster, there are four groups (G3, G4, G5, and G6) where all the minority varieties are grouped along with Syrah and Merlot among the traditional ones.

In both white and red grape varieties, it is noteworthy that the primary differentiation between these groups primarily hinges on acids, except for hexanoic acid, and esters, except for ethyl decanoate. The heightened concentration of these compounds may be attributed to the genetic adaptation of these minority grape varieties to the specific region. This genetic adaptation could potentially activate distinct metabolic pathways, resulting in the increased accumulation of these compounds as part of their adaptive response. Hence, this grouping implies that the recovered varieties under examination could yield wines with an aromatic profile distinct from those produced with the traditional varieties of the region.

The identified compounds were grouped into acids, alcohols, esters, norisoprenoids, aldehydes, and terpenes. Acids were represented by octanoic, hexanoic, and decanoic acid. Although the sensorial contribution of these compounds is related with unpleasant odor descriptors such as rancid or cheese [20], some authors [21] suggest that they contribute to wine’s fresh flavor and they also help to modify the perception of other taste sensations. In the case of white grapes (Figure 1), varieties of group 1 would be the most abundant, followed by groups 4 and 5. Thus, within these, the most acidic variety was Macabeo under irrigation, where its concentration of octanoic acid stood out; however, in rainfed conditions, the content of this compound decreased significantly. The second most acidic variety was Chardonnay in both regimes, although in this case the most abundant compound was decanoic acid, mainly in rainfed conditions. Similarly, if we focus on the recovered varieties, Maquías and Mizancho (G5) were the most acidic, but unlike the traditional ones, the most abundant compound was hexanoic acid, although in lower concentrations than the previous ones, which was positively affected (increased) by irrigation in the Maquías variety and negatively affected (decreased) in Mizancho. In red grapes, the traditional varieties Garnacha, Tempranillo, and Bobal (G1 and G2) showed the highest concentration of these compounds. Although some authors did not observe an effect on the concentration of these compounds by water status in the vineyards [22] this study highlighted the total levels in the Tempranillo variety under irrigation, which exhibited concentrations almost ten times higher than the rest of the varieties. On the other hand, the recovered varieties presented very low concentrations of these compounds.

Regarding alcohols, in the case of white grapes, varieties of groups G2 and G3 showed the highest concentration of these, with 1-hexanol and 2-hexen-1-ol being the two most abundant compounds. Among all the varieties, those that presented a higher concentration of these compounds were the minority ones, such as Blanca del Tollo and Jarrosuelto, being negatively affected by irrigation. This behavior has already been described previously by other authors, who suggested that low water availability increased the concentration of some C6 compounds [22,23],. However, this trend was not as clear in all varieties of this study, since, among the recovered varieties, Pintada and Azargón showed higher concentrations under irrigation conditions. In the case of red grapes, the varieties in groups 5 and 6 showed the highest levels of these compounds, with the highest levels of 1 hexanol and 2-hexen-1-ol in Tinto Velasco, regardless of the water regime, and the 3 hexen-1-ol and benzyl alcohol content in the varieties Moravia Agria and Tinto Fragoso in irrigation conditions, respectively. Thus, of the traditional varieties, the presence of phenylethyl alcohol in the Tempranillo variety under irrigation conditions was of note. In terms of the contribution to the aromatic profile of wines of this group of compounds, C6 alcohols are considered to be responsible for the herbaceous aroma of wines [24] but 2-phenylethyl alcohol is an aromatic alcohol that contributes with rose notes to the wine [25].

Terpenes are one of the most important compounds in wine, which contribute to the varietal characteristics of wine thanks to their flowery and sweet aroma nature [26,27] and, in this work, linalool, geraniol, and geranyl acetone were identified. In the case of white varieties (Figure 1), linalool was only detected in the Riesling and Moscatel Serrano varieties, while the latter two (geraniol and geranyl acetone) were found in the remaining varieties. As expected, the most terpene-rich varieties were Moscatel Serrano and Riesling (G4 and G5), attributed to the presence of linalool, which was not found in the rest of the varieties. The concentration of these compounds in the other recovered varieties was very similar, except in Blanca del Tollo, where only geranyl acetone was identified. Moreover, the highest concentration of terpenes in these varieties was observed in Jarrosuelto under irrigation conditions. In terms of the effect of the water supply, despite some authors suggesting that controlled water stress triggers specific biochemical responses favoring terpene synthesis [14,19] this behavior was only observed in the Albillo Dorado and Chardonnay varieties, with even the Mizancho variety decreasing terpene content under water deficit. Therefore, the fact that these recovered varieties show similar or even higher concentrations of terpenes compared to some traditional varieties (Macabeo or Airén), regardless of the water regime, indicates that they could give rise to wines with distinctive and complex aromatic profiles. On the other hand, concerning red grapes (Figure 2), generally the most terpene-rich varieties were found within groups 4, 5, and 6. In this case, the effect of water availability was observed. Thus, the highest concentration of geraniol was found in Tinto Fragoso under irrigation conditions, followed by Tinto Velasco and Moravia Agria, but in this instance under rainfed conditions. Also, among the recovered varieties, the content of geranyl acetone stood out in the Benedicto variety under irrigation conditions. Finally, regarding linalool, Tinto Velasco showed the highest content among the recovered varieties, although the content in Garnacha Tinta (G1) under irrigation conditions is noteworthy.

Ester compounds have been positively associated in sensory studies with the overall aroma of the wine, including red and dark fruits, among others. In this work, this group of compounds was the most affected by a water regime, showing lower concentrations of these compounds under rainfed conditions, except for the Chardonnay and Moscatel Serrano varieties. These results are consistent with the results obtained by Talaverano et al. [28], who suggested that water-restricted treatment negatively affects the fatty acid metabolism from which these esters are derived. Among the minority varieties, Jarrosuelto, Azargón, and Maquías showed the highest amount of these compounds. In the case of red grapes, the varieties with the highest content of these compounds were the traditional Bobal and Tempranillo, mainly under irrigation conditions. Among the minority varieties, a higher content of ethyl decanoate was observed in the Tinto Velasco variety, regardless of the water regime.

Norisoprenoids represent key odoriferous compounds in wine, most of which are characterized by floral and fruity pleasant notes [29] and were represented by β-ionone and β-damascenone in this work. In white grapes (Figure 1), among the minority varieties studied, Mizancho and Maquías were the ones that showed a higher concentration of these compounds, in line with the values obtained for the Riesling variety. On his part, in the case of red grapes (Figure 2), the varieties of group 6 (Tortozona Tinta, Moribel, and Tinto Fragoso (irrigation)) had the highest concentration of these compounds, with similar ionone levels in all of them and damascenone standing out in Tortozona Tinta in rainfed conditions.

Finally, aldehydes were represented by nonanal, benzaldehyde, and hexenal. In the case of white grapes, varieties of group 5 showed the highest concentration of these compounds, with 2-hexenal standing out, mainly due to its high concentration in the variety Maquías in rainfed conditions. Regarding red grapes, the highest concentrations of these compounds were observed in groups 5 and 6, indicating that the recovered varieties have a higher amount of these compounds. Thus, the concentration of hexenal in Tortozona Tinta, in both regimes, was the highest of all.

To determine the effect of the three main factors considered in this work (extract pH, water regime, and grape variety) on the volatile composition of grapes, as well as the groups of compounds that are most affected by these factors, a multivariate analysis (MANOVA) was performed (Table S1). Among the individual factors, extract pH and grape variety had the most significant effect in alcohols, aldehydes, and norisoprenoids groups of compounds.

**Figure 3:**
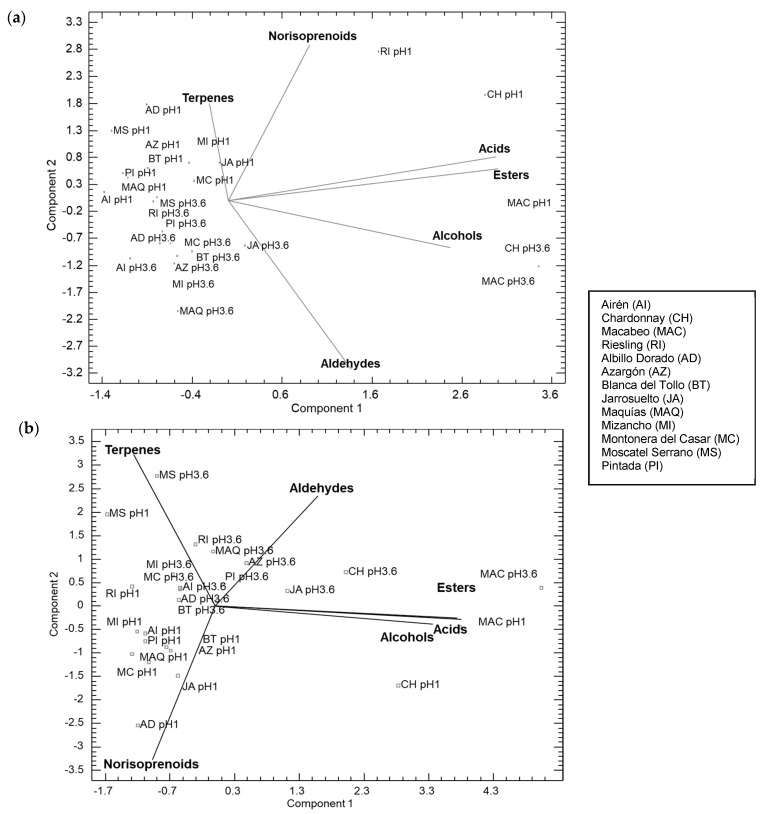
PCA with the white grapes’ aromatic compound groups at extract pH 1 and pH 3.6 and two water regimes: (**a**) rainfed conditions; (**b**) irrigation conditions.

**Figure 4:**
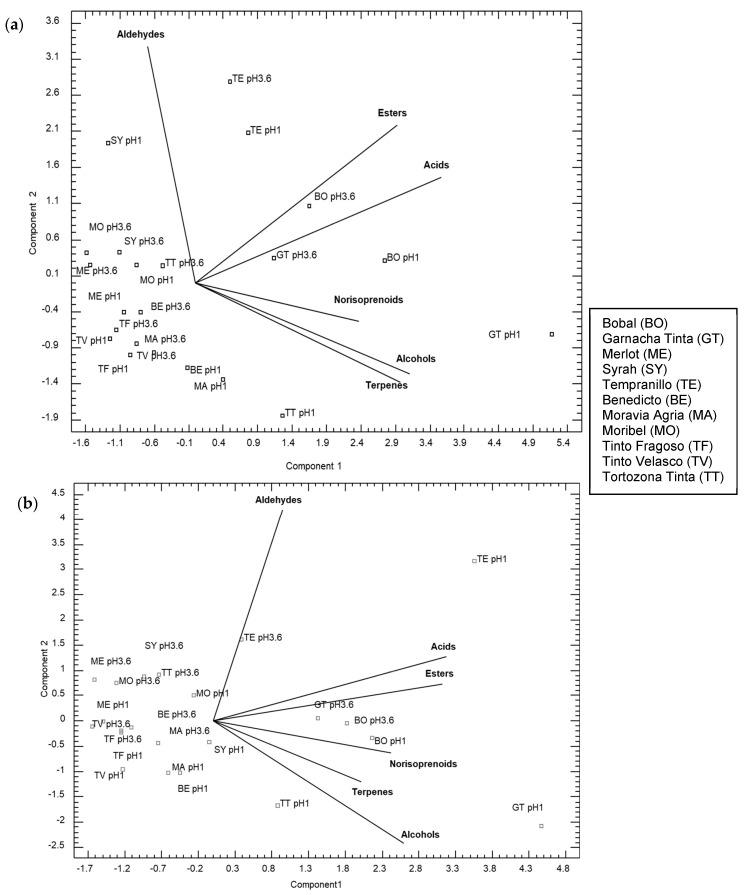
PCA with the red grapes’ aromatic compound groups at extract pH 1 and pH 3.6 and two water regimes: (**a**) rainfed conditions; (**b**) irrigation conditions.

Finally, with the purpose of obtaining an overall view of the total content of aroma compounds, not only the easily extractable, a principal component analysis was performed with the total content of aromatic compounds of the extracts obtained at both pHs (1 and 3.6), in white and red varieties and with both water regimes (Figure 3 and Figure 4). Compounds were grouped into terpenes, norisoprenoids, acids, esters, alcohols, and aldehydes. So, lines of the compound groups indicate a higher concentration of compounds as we move further away from the point where the lines intersect.

In relation to Figure 3, in both water regimes, a clear separation was observed in component 1 between the traditional Macabeo and Chardonnay varieties and those recovered, indicating the aromatic disparity between the two varieties. Thus, in Figure 3a, when moving to component 2, it becomes evident that the extracts obtained at pH 1 presented a greater concentration of terpenes and norisoprenoids, highlighting the disparity in the Riesling variety between the two regimes. These suggest a more efficient extraction of aromatic compounds from grapes under acidic conditions, thereby justifying the higher extractability observed at pH 1 compared to neutral or basic extraction conditions. On the other hand, in Figure 3b, focusing on the negative part of component 2, it is noticeable that the extracts obtained at pH 1 were characterized by an increase in the content of norisoprenoids. These results suggest that, in the recovered white varieties, dry conditions favor the enhancement of aromatic precursors of terpenes and norisoprenoids. This implies that the wines obtained could have a richer aromatic profile under water stress conditions and, therefore, confirms that they could be a good alternative for cultivation in areas with water scarcity.

Regarding red varieties (Figure 4), the effect of extract pH on the increase in the content of certain groups of compounds was not as clear. Thus, under rainfed conditions (Figure 4a), a significant separation was observed in component 1 between the traditional varieties Garnacha, Bobal, and Tempranillo, and the recovered minority varieties. On the other hand, when shifting to component 2, it can be observed that the Tempranillo variety, a reference in Spanish wines, had a less aromatic profile in terms of terpenes and norisoprenoids compared to other traditional varieties. Finally, under irrigation conditions, a clear separation is observed in the extracts at pH 1 of the Tempranillo and Garnacha Tinta varieties, with these showing a higher content of all the studied compound groups.

## 3. Materials and Methods

### 3.1. Grapevines and Water Regime

Grapes were sampled in 2021 from a multivarietal experimental vineyard located in the Regional Institute of Agri-Food and Forestry Research and Development of Castilla-La Mancha (IRIAF), in Tomelloso, Castilla-La Mancha, Spain (latitude 39°10′14″ N, longitude 3°00′16″ W; altitude 660 m.a.s.l.). The climate of the area is continental semi-arid Mediterranean. The soil of the vineyard was Petric Calcisol (FAO soil classification) or Petrocalcic Calcixerept (USDA soil classification) with loam texture and active limestone, and organic matter contents of 15% and 3.2%, respectively. Soil depth was 30 cm below which there was a petrocalcic horizon, impenetrable by the vine roots. This type of soil, widely represented in La Mancha wine region, is traditionally associated with grapevine growing. The pedoclimatic soil conditions are characterized by the typical xeric moisture regime of Mediterranean climates. The vineyard was planted in 2008 and grafted onto Fercal rootstock. The vines were planted at 2.8 m between rows and 1.2 m between vines (2976 vines/ha) and were trained using the VSP trellis system and the bilateral Royat cordon formation. For the study, 9 widespread varieties (5 red and 4 white) and 15 minority/recovered ones (6 red and 9 white) were selected (Table 2).

For each variety, 50 vines were studied and subjected to two different water-deficit regimes: 20 vines were grown under rainfed conditions with survival irrigation (rainfed treatment, 273 m^3^/ha net supplied), and 30 vines were grown under irrigation (irrigation treatment, 977 m^3^/ha net supplied). To determine the harvest date, regular sampling was conducted until the grapes reached a concentration of total soluble solids between 22.5 and 24.5°Brix for red cultivars, and between 20 and 22°Brix for white cultivars. At that time, a representative quantity of grapes of about 1.5 kg from each water treatment was harvested and frozen for subsequent analysis.

### 3.2. Grape-Extraction Procedure

For the extraction, 200 berries randomly selected were crushed and homogenized at 4000 r.p.m. Then, 50 g of the mixture was weighed in two centrifuge flasks and 50 mL of pH 3.6 aqueous solution (5 g/L tartaric acid adjusted to pH 3.6 with NaOH) was added to one and 50 mL of pH 1 aqueous solution (HCl 0.1 N at pH 1) to the other. The solutions were homogenized and macerated for 24 h at room temperature (21 °C ± 3) and after centrifugation at 4000 rpm for 10 min the limpid supernatant was recovered. Two extracts were obtained (pH 1: total extraction potential; and pH 3.6: easily extractable compounds) on which analytical determinations were performed.

### 3.3. Chemical Analysis

#### 3.3.1. Volatile Compounds

Volatiles were determined according to Sánchez-Gómez et al. [30]. Briefly, the extraction was carried out by means of Headspace Stir bar Sorptive Extraction (HS-SBSE) with a polydimethylsiloxane twister bar (10 mm length, 0.5 mm film thickness). A total of 22 mL of grape extracts were placed in a 50 mL vial and a polydimethylsiloxane coated stir bar (twister, 0.5 mm film thickness, 10 mm length; Gerstel, Mülheim, and der Ruhr, Germany) was inserted into the twister headspace vial and hermetically closed. The vial was stirrer for 1 h at 500 rpm with a temperature of 60 °C. Later, analysis was performed using an automated thermal desorption unit (TDU, Gerstel, Mülheim and der Ruhr, Germany) mounted on an Agilent 7890A gas chromatograph system (GC) coupled to a quadrupole Agilent 5975C electron ionization mass spectrometric detector (MS, Agilent Technologies, Palo Alto, CA, USA) equipped with a fused silica capillary column (BP21 stationary phase; 30 m length; 0.25 mm I.D.; and 0.25 μm film thickness) (SGE, Ringwood, Australia). The carrier gas was helium with a constant column pressure of 20.75 psi.

The Stir Bars were thermally desorbed in a stream of helium carrier gas at a flow rate of 75 mL/min with the TDU programmed from −10 °C to 295 °C (held 5 min) at a rate of 60 °C/min at splitless desorption mode. The analytes were focused on a programmed temperature vaporizing injector (PTV) (CIS-4, Gerstel), containing a packed liner (20 mg tenax TA), held at −10 °C with cryo cooling prior to injection. After desorption and focusing, the CIS-4 was programmed from −10 °C to 260 °C (held for 5 min) at 12 °C/min to transfer the trapped volatiles onto the analytical column. The GC oven temperature was programmed to 40 °C (held for 2 min), raised to 80 °C (5 °C/min, held for 2 min), raised to 130 °C (10 °C/min, held for 5 min), raised to 150 °C (5 °C/min, held for 5 min), and then raised to 230 °C (10 °C/min, held for 5 min). The MS was operated in scan acquisition (27–300 m/z) with an ionization energy of 70 eV. The temperature of the MS transfer line was maintained at 230 °C. MS data acquisition was carried out at positive scan mode, although to avoid matrix interferences, the MS quantification was performed in the single ion-monitoring mode using their characteristic m/z values. Information related to analyzed compounds and m/z values are included in greater detail in Sánchez-Gómez et al.’s [18] work. Compounds identification was performed using the NIST library and confirmed via comparison with the mass spectra and retention time of their pure standards (Sigma-Aldrich, Steinheim, Germany). As internal standard, 3-methyl-1-pentanol was used. Quantification was based on calibration curves of the respective standards at five different concentrations (R^2^ = 0.95–0.97). The analyses of each replicate of grape extracts were conducted in triplicate.

#### 3.3.2. Varietal Aroma Potential Index

The varietal aroma potential index (IPAv) of grapes was analyzed using a commercially available IPAv kit (Teknokroma, Barcelona, Spain) according to Serrano de la Hoz et al. [31] The method, based on Salinas et al. [32], is a spectrophotometric determination of the glucose released from the glycosylated aroma precursors via acid hydrolysis. Samples were analyzed in triplicate.

#### 3.3.3. Statistical Analysis

Heatmaps were performed using MetaboAnalyst 5.0 Web service. Raw data were normalized by median and auto scaled (mean-centered and divided by standard deviation of each variable) prior to the analysis. Moreover, one-way analysis of variance (ANOVA) at a 95% probability level, according to the Fisher post-hoc test, was used to determine the differences between the IPAv grapes values. Finally, a multivariate analysis of variance (MANOVA) and a principal component analysis (PCA) were performed. These analyses were conducted using the Statgraphics Centurion statistical program (version 18.1.12; StatPoint, Inc., The Plains, VA, USA).

## 4. Conclusions

The results of this work showed significant variations among cultivars and water-management approaches investigated, generally indicating elevated levels of aromatic compounds in the rainfed regime. Some recovered minority varieties such as Maquías, Moscatel Serrano, Tinto Fragoso, and Tortozona Tinta stood out, which, grown under low water availability conditions, showed a similar or even more suitable aromatic composition in comparison with grapes of other varieties whose cultivation is more widespread, such as Chardonnay, Merlot, and Syrah. This suggests potential adaptability to forthcoming climate change conditions in these specific scenarios.

## Figures and Tables

**Table 1:** Varietal aroma potential index (IPAv) of red and white grapes.

	Rainfed	Irrigation	*p*
***Red varieties***			
Bobal	10.83 ± 0.11	**12.14 ± 0.06**	***
Garnacha Tinta	**18.37 ± 0.06**	14.50 ± 0.08	***
Merlot	**10.90 ± 0.04**	10.12 ± 0.30	*
Syrah	7.46 ± 0.02	**8.79 ± 0.15**	***
Tempranillo	**14.06 ± 0.01**	9.23 ± 0.02	***
Benedicto	11.73 ± 0.03	**16.67 ± 0.02**	***
Moravia Agria	**7.31 ± 0.08**	6.88 ± 0.02	***
Moribel	**11.83 ± 0.01**	9.49 ± 0.01	***
Tinto Fragoso	8.22 ± 0.04	**9.92 ± 0.24**	***
Tinto Velasco	**13.24 ± 0.06**	8.48 ± 0.06	***
Tortozona Tinta	4.38 ± 0.02	**5.43 ± 0.06**	***
***White varieties***			
Airén	7.93 ± 0.02	**19.53 ± 0.31**	***
Chardonnay	**5.97 ± 0.03**	4.88 ± 0.02	***
Macabeo	9.51 ± 0.17	**14.85 ± 0.09**	***
Riesling	12.02 ± 0.04	**15.71 ± 0.08**	***
Albillo Dorado	17.61 ± 0.14	**21.76 ± 0.26**	***
Azargón	13.69 ± 0.06	**14.54 ± 0.11**	***
Blanca del Tollo	7.98 ± 0.02	7.66 ± 0.02	***
Jarrosuelto	6.41 ± 0.08	**6.54 ± 0.02**	*
Maquías	**7.69 ± 0.04**	6.58 ± 0.00	***
Mizancho	8.34 ± 0.01	**9.26 ± 0.07**	***
Montonera del Casar	5.76 ± 0.04	**7.19 ± 0.04**	***
Moscatel Serrano	12.08 ± 0.09	**20.51 ± 0.06**	***
Pintada	7.36 ± 0.06	**11.96 ± 0.09**	***

For each variety, differences between water regimes are indicated according to Fisher’s LSD test. The highest values are in bold. * *p* value < 0.05; ** *p* value < 0.01; *** *p* value < 0.001. Bold values indicate the highest value within a variety.

**Table 2:** Studied varieties.

White		Red	
Reference	Airén (AI)	Reference	Bobal (BO)
	Chardonnay (CH)		Garnacha Tinta (GT)
	Macabeo (MAC)		Merlot (ME)
	Riesling (RI)		Syrah (SY)
Minority/recovered	Albillo Dorado (AD)		Tempranillo (TE)
	Azargón (AZ)	Minority/recovered	Benedicto (BE)
	Blanca del Tollo (BT)		Moravia Agria (MA)
	Jarrosuelto (JA)		Moribel (MO)
	Maquías (MAQ)		Tinto Fragoso (TF)
	Mizancho (MI)		Tinto Velasco (TV)
	Montonera del Casar (MC)		Tortozona Tinta (TT)
	Moscatel Serrano (MS)		
	Pintada (PI)		

## Data Availability

Data are contained within the article and supplementary materials.

## Supplementary Materials

The following supporting information can be downloaded at: https://www.mdpi.com/article/10.3390/plants13111507/s1, Table S1. Total rainfall and monthly mean temperature for the experiment in the 2021 agronomic year. Table S2. Length and date of each phenological stage in the different varieties. Table S3. MANOVA of white and red grapes. Factors: pH, water regime and grape variety; Table S4. Component Weights for Figure 3 and Figure 4.
