# Supplementary material for: Volatile Characterization of Recovery Minority Grape Varieties from Castilla-La Mancha Region (Spain)
Source: Plants (Basel). 2024 May 30;13(11):1507. doi: 10.3390/plants13111507 (PMC11174939; doi:10.3390/plants13111507)
Supplement: Supplementary file 1 [file plants-13-01507-s001.zip › plants-2991708-supplementary.pdf]

SUPPLEMENTARY MATERIAL

Table S1. Total rainfall and monthly mean temperature for the experiment in the 2021 agronomic year.

2021	Oct.	Nov.	Dec.	Jan.	Feb.	Mar.	Apr.	May	Jun.	Jul.	Aug.	Sep.
Tm (ºC)	13.77	10.85	6.18	4.35	9.82	9.89	12.74	18.26	23.08	26.80	27.03	21.13
R (mm)	27.00	45.40	40.70	60.50	28.60	4.60	56.10	6.00	39.90	0.40	2.00	16.00
T (ºC)	21.07	17.43	10.92	9.43	16.17	17.24	19.03	26.12	30.38	34.81	35.27	28.43
t (ºC)	6.93	5.82	1.89	0.33	4.59	2.80	7.24	10.11	15.14	17.39	18.14	14.07

Tm: mean temperature; R: mean rainfall; T: mean of maximums temperature; t: mean of minimums temperature. Source: weather station located within the study plot.

Table S2. Length and date of each phenological stage in the different varieties.

Variety	Budbreak	Flowering	Veraison	Maturity
Benedicto	12-apr.	3-jun.	7-aug.	2-sep.
Bobal	9-apr.	30-may	28-jul.	10-sep.
Garnacha Tinta	4-apr.	31-may	6-aug.	13-sep.
Merlot	7-apr.	30-may	3-aug.	23-aug.
Moravia Agria	7-apr.	2-jun.	7-aug.	30-sep.
Moribel	14-apr.	2-jun.	8-aug.	3-sep.
Syrah	7-apr.	30-may	29-jul.	24-aug.
Tempranillo	6-apr.	30-may	25-jul.	20-aug.
Tinto Fragoso	6-apr.	31-may	30-jul.	30-aug.
Tinto Velasco	9-apr.	30-may	4-aug.	23-sep.
Tortozona Tinta	9-apr.	1-jun.	12-aug.	6-sep.
Airén	12-apr.	5-jun.	2-aug.	15-sep.
Albillo Dorado	14-apr.	2-jun.	22-jul.	4-aug.
Azargón	5-apr.	2-jun.	8-aug.	27-aug.
Blanca del Tollo	8-apr.	31-may	26-jul.	20-aug.
Chardonnay	3-apr.	29-may	28-jul.	13-aug.
Jarrosuelto	21-apr.	6-jun.	3-aug.	26-aug.
Macabeo	12-apr.	1-jun.	9-aug.	7-sep.
Maquías	3-apr.	31-may	2-aug.	17-aug.
Mizancho	11-apr.	30-may	31-jul.	18-aug.
Montonera del Casar	10-apr.	30-may	24-jul.	16-aug.
Moscatel Serrano	31-mar.	31-may	1-aug.	18-aug.
Pintada	5-apr.	30-may	29-jul.	19-aug.
Riesling	6-apr.	29-may	6-aug.	27-aug.

Table S3. MANOVA of white and red grapes. Factors: pH, water regime and grape variety.

	pH	Water regime (Hr)	Grape variety (Gv)	pH x Wr	pH x Gv	Wr x Gv	pH x Wr x Gv
Alcohols	0.06		**		***		
Aldehydes	***				*		
Esters							
Terpenes							
Norisoprenoids	***						
Acids							

** p value < 0.05; ** p value < 0.01; *** p value < 0.001*

Table S4. Component Weights for Figures 3 and 4.

a) White grapes rainfed

	Component 1 (40.42%)	*Component 2 (22.45%)*
Alcohols	0.409513	-0.314554
Aldehydes	0.110761	-0.707071
Esters	0.612995	0.0180794
Terpenes	0.0859962	0.193415
Norisoprenoids	0.250957	0.600986
Acids	0.611468	0.0467609

b) White grapes irrigation

	Component 1 (41.13%)	*Component 2 (22.50%)*
Alcohols	0.452012	0.195566
Aldehydes	0.244286	-0.45777
Esters	0.592665	0.0759325
Terpenes	-0.178412	-0.563537
Norisoprenoids	-0.163338	0.654331
Acids	0.571181	-0.026679

c) Red grapes rainfed

	Component 1 (40.50%)	*Component 2 (21.30%)*
Alcohols	0.459061	-0.273039
Aldehydes	-0.102971	0.708868
Esters	0.431823	0.470922
Terpenes	0.438554	-0.298216
Norisoprenoids	0.350023	-0.114467
Acids	0.526634	0.314885

d) Red grapes irrigation

	Component 1 (41.47%)	*Component 2 (20.56%)*
Alcohols	0.424413	-0.462833
Aldehydes	0.510526	0.138586
Esters	0.330236	-0.230429
Terpenes	0.396104	-0.121107
Norisoprenoids	0.518877	0.242199
Acids	0.155076	0.800096
